# Different Neural Mechanisms for the Comparison and Priming Distance Effects: An fMRI Study

**DOI:** 10.3389/fpsyg.2016.01633

**Published:** 2016-10-26

**Authors:** Li Zhang, Fei Cai, Chuansheng Chen, Qinghua He

**Affiliations:** ^1^Department of Psychology, Southwest UniversityChongqing, China; ^2^Department of Psychology and Social Behavior, University of California, Irvine, IrvineCA, USA; ^3^Key Laboratory of Mental Health, Institute of Psychology, Chinese Academy of SciencesBeijing, China; ^4^Southwest University Branch, Collaborative Innovation Center of Assessment Toward Basic Education Quality at Beijing Normal UniversityChongqing, China

**Keywords:** distance effect, comparison distance effect, priming distance effect, meta-analysis, fMRI

## Abstract

Using functional magnetic resonance imaging, the present study examined whether the comparison distance effect (CDE) and the priming distance effect (PDE) in number processing had the same underlying neural mechanisms. 24 healthy participants completed a number comparison task and a number priming task in the scanner. Imaging data were examined for brain regions selected based on a meta-analysis of previous studies of number processing. Results revealed robust CDE and PDE at both behavioral and neural levels. The CDE had a significant hemodynamic signature in the right parietal cortex but not in the left parietal cortex, although a direct test of this hemispheric laterality did not reach statistical significance. In contrast, the PDE showed significant left-hemisphere laterality with a significant hemodynamic signature in the left parietal cortex but not in the right parietal cortex. These results suggested that the CDE and PDE had different underlying neural mechanisms.

## Introduction

One of the most well-documented phenomena in number processing is the distance effect (DE). This effect was first observed by Moyer and Landauer (1967) in a numerical comparison task. The comparison distance effect (CDE) refers to the fact that it takes less time to compare number pairs that are farther apart (e.g., 1 and 9) than those that are closer to each other (e.g., 2 and 3). The CDE has been found in various number formats (i.e., dot patterns, Arabic numbers, number words, etc.). In addition to the CDE, another distance effect was later observed in priming studies. The priming distance effect (PDE) refers to faster responses to targets when they are preceded by a numerically closer prime (e.g., Reynvoet et al., 2002; Notebaert et al., 2010). For example, the digit “5” is processed faster when preceded by “4” than by “9”.

Traditionally, researchers have used the mental number line to explain both CDE and PDE (e.g., Restle, 1970; Dehaene, 1997). According to this perspective, numbers are represented in the brain along a line analogous to the physical number line on paper. Mental numbers are also represented as distributions, which have greater overlaps among neighboring numbers (i.e., close distance) than among numbers at a distance from one another (i.e., far distance). The CDE is observed because overlapping representations are more difficult to discriminate than non-overlapping ones, whereas the PDE is observed because the preceding prime already partially activated the target representation. Accordingly, the CDE (e.g., De Smedt et al., 2009; Holloway and Ansari, 2009) and PDE (e.g., Reynvoet et al., 2002; Notebaert et al., 2010) have been used as indicators of the processing of numerical magnitude.

However, at least two recent studies (Van Opstal et al., 2008; Sasanguie et al., 2011) have directly challenged the notion that the CDE and PDE share the same mechanism. Specifically, Van Opstal et al. (2008) used letter and number stimuli in a number priming experiment and found that both number and letter comparisons showed the CDE, but only number comparison showed the PDE. Using non-symbolic number stimuli (dots), Sasanguie et al. (2011) also found that the CDE was not correlated with the PDE. In addition, evidence from developmental studies has also shown a dissociation between the CDE and PDE: Whereas the CDE decreases with age (e.g., Sekuler and Mierkiewicz, 1977; Holloway and Ansari, 2009), the PDE does not show developmental changes (Reynvoet et al., 2009; Defever et al., 2012). This debate needs to be resolved for both theoretical and practical reasons. Theoretically, a common neural substrate for both CDE and PDE would further support the importance of the mental number line in number processing. Practically, if the CDE and PDE share a common mechanism, either of them can be used to predict general math ability. Otherwise, both the CDE and PDE need to be considered.

To help address the question of whether the CDE and PDE originate from the same mechanism, we conduct a slow event-related functional magnetic resonance imaging (fMRI) study. The slow event-related fMRI design allowed us to track the time course of all trials and to probe whether and when significant differences between the CDE and PDE appeared in the brain responses. To our knowledge, this is the first brain imaging study to explicitly test the underlying neural mechanism of both CDE and PDE. We focused our analyses on the parietal lobe because of its crucial role in number processing (e.g., Dehaene et al., 2003; Eger et al., 2003; Fias et al., 2003; Cantlon et al., 2006; Piazza et al., 2007; Dormal and Pesenti, 2009; Nieder and Dehaene, 2009). To identify specific brain areas for number processing, we first conducted a meta-analysis of previous studies using number processing and calculation tasks. It should be noted that it would have been ideal to contrast brain areas for the CDE with those for the PDE, but there were very few neuroimaging studies of the PDE (Notebaert et al., 2010; Sasanguie et al., 2013).

## Materials and Methods

### Participants

Twenty-four right-handed college students (14 females, mean age = 21.29 years, *SD* = 1.55, and age range: 19–25 years) participated in the present study. They were recruited through flyers posted across the campus of Southwest University, China. All of them had normal or corrected-to-normal vision and had no history of psychiatric or neurological disorders based on self-report. Written informed consent was obtained from each participant, and this study was approved by the Administration Committee of Psychological Research at Southwest University.

### Tasks

Arabic numbers 1–9 were used as stimuli in both comparison and priming tasks. Similar to the study by Notebaert et al. (2010), number pairs were divided into small-distance pairs (those that differed by 1) and large-distance pairs (those that differed by 4, 5, or 6) to probe the distance effect. Three different distances (4, 5, and 6) were included in the large-distance pairs in order to create enough number pairs (16 pairs each for the small- and large-distance conditions). The numbers 1 and 9 were never used as the first numbers in order to avoid situations in which participants would be able to make number comparisons after only seeing the first number without the need to see the second number.

**FIGURE 1:**
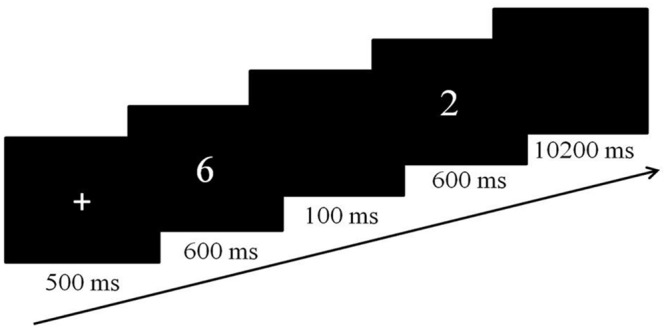
**The experimental flow of a single trial for both experimental tasks**.

In both tasks, a slow event-related design was used and each trial started with a fixation cross, presented centrally for 500 ms (**Figure 1**). The fixation cross was then replaced by two sequentially presented numbers at the same location for 600 ms each, separated by a blank screen of 100 ms. When the second number disappeared from the screen, a blank screen was presented for an additional 10200 ms, resulting in an event length of 12 s. Both tasks included four runs of 6 min 34 s each. Each run consisted of the same 32 trials. The order of the tasks was counterbalanced across participants. Participants were asked to respond as quickly and accurately as possible for both tasks.

In the comparison task, participants were asked to press the left button when the first number was larger and press the right button when the second number was larger.

The priming task was adapted from Notebaert et al. (2010), in which participants were informed of a predefined number. The predefined number was always one of the four numbers (2, 3, 7, or 8) and was presented on the instruction screen as an Arabic numeral at the start of each run. For each participant the predefined four numbers were used in a random order. Then participants were shown the priming number (the first number) followed by a probe number (the second number), and asked to judge whether the probe number matched the predefined number. They were instructed to press the right button when the probe number matched the predefined number, which occurred in 25% of the trials.

### Procedure and Data Acquisition

Participants lay supine on the fMRI scanner bed, and viewed the task back-projected onto a screen through a mirror attached onto the head coil. Foam pads were used to minimize head motion. Stimulus presentation and timing of all stimuli and response events were achieved by using E-prime 2.0 (Psychology Software Tools, Inc. Pittsburgh, PA, USA) on a windows PC. Participants’ responses were collected online using an MRI-compatible button box. The fMRI images were acquired with a Siemens 3T scanner (Siemens Magnetom Trio TIM, Erlangen, Germany) at the Brain Imaging Center of Southwest University. Each functional run involved the acquisition of 197 EPI volumes (slices = 32, TR/TE = 2000 ms/30 ms, flip angle = 90°, FOV = 220 × 220 mm^2^, matrix size = 64 × 64, slice thickness = 3 mm, and slice gap = 1 mm). After the participants completed 8 functional runs, three-dimensional high-resolution T1 anatomical images were recorded with a total of 176 slices at a thickness of 1 mm and in-plane resolution of 0.98 × 0.98 mm (TR/TE = 1900 ms/2.52 ms, flip angle = 90°, and FOV = 256 × 256 mm^2^).

### Procedure of the Meta-Analysis

Two databases (the PubMed and the Web of Science) were used to search for neuroimaging studies of number processing (with the keywords “fMRI” and “number processing”) published before March 25, 2014. The search yielded 2666 articles, but 2467 of them were completely irrelevant to the topic of number processing. Of the remaining 199 studies, 58 (Supplementary Table S1) were selected for our meta-analysis according to the following criteria: (i) none were case studies, reviews, or meta-analysis studies; (ii) they used whole-brain scanning and reported complete coordinates of the activation instandardized stereotaxic space, such as the Talairach atlas or the MNI (Montreal Neurological Institute) atlas; and (iii) stimuli were positive integers. It should be noted that for studies comparing normal controls with psychiatric or neurological patients, only data from normal controls were included. In addition, to guarantee the statistical independence between the estimated experimental effects, only one contrast most relevant to number processing from each independent group in each identified study (Supplementary Table S1) was included in the meta-analysis (Arsalidou and Taylor, 2011; Kaufmann et al., 2011; Sescousse et al., 2013).

The meta-analysis was performed using GingerALE (the activation likelihood estimation; version 2.3.1)^1^, which generated regions of a reliable activity across all selected contrasts (Laird et al., 2005; Eickhoff et al., 2009, 2012; Turkeltaub et al., 2012). In the current study, the coordinates of the local maxima for all foci activated for the selected contrast were extracted. Foci reports in Talairach space were first converted to MNI space using the built-in transformation algorithms in GingerALE. All coordinates were then pooled into GingerALE to perform the activation likelihood estimation. The output probabilistic maps of activation were thresholded at *p* < 0.01, corrected for multiple comparisons using the false discovery rate and with a minimum cluster volume of 270 mm^3^ (corresponding to 10 voxels).

### Data Analysis

#### Behavioral Data Analysis

Only response trials were analyzed in a paired *t*-test with the distance between the prime and the probe (small vs. large) as the independent factor to test whether there was a significant PDE at the behavioral level in the priming task. Consistently, in the comparison task, only trials with the same stimuli were used to explore the CDE.

#### Image Processing and Statistical Analysis

For the priming task, imaging data analysis used the trials that did not require participants’ responses to avoid a potential contamination of response-related processes. Consistently, in the comparison task, only trials with the same stimuli were used to explore the neural CDE. No participant was excluded due to head motion. Image preprocessing and statistical analysis were carried out by using FEAT (fMRI Expert Analysis Tool), part of the FSL (FMRIB’s software library)^2^. After motion correction, functional images were spatially smoothed using a 5-mm full-width-half-maximum (FWHM) Gaussian kernel and temporally high-pass filtered with a cut-off period of 100-s. The blood oxygen level dependent (BOLD) response was modeled separately for each trial. The event onsets (when the second number was presented) were convolved with a canonical hemodynamic response function (HRF, double-gamma function) to generate regressors used in the general linear model (GLM). Temporal derivatives were included as covariates of no interest to improve statistical sensitivity. Data were then fitted to the model using FSL’s implementation of the general linear model. A two-step registration procedure was used whereby functional images were first registered to the three-dimensional high-resolution structural image, and then into the standard MNI space, using affine transformations (Jenkinson and Smith, 2001). The registration from the functional–structural image to the standard space was further refined using FNIRT non-linear registration. Next, we extracted the time course for each trial using PEATE (Perl Event-related Average Timecourse Extraction)^3^ with 2 s before each event as the baseline. Analysis was limited to the regions of interests (ROIs) identified by the meta-analysis. Finally, the activation level (% signal change) was extracted from all trials across 12 s and an average time course was then obtained for each ROI and each condition (small vs. large distance) and task (comparison vs. priming).

## Results

### Behavioral Results

Due to a technical error, only 17 participants had valid behavioral data. Because of their high accuracy on both tasks (average accuracy = 99%), we only analyzed the reaction time data from the correct trials. A repeated-measure ANOVA with distance and task as within-subject factors was conducted. Results revealed a significant main effect of task, *F*(1,16) = 29.662, *p* < 0.001, and a significant task × distance interaction, *F*(1,16) = 47.699, *p* < 0.001. The simple effect analyses showed that the interaction was due to a significant CDE for the comparison task, *F*(1,16) = 43.314, *p* < 0.001, with longer RTs for the small distance (655 ± 148 ms) than for the large distance (622 ± 140 ms). In contrast, a significant PDE was found for the priming task, *F*(1,16) = 43.314, *p* < .001, with shorter RTs for the small distance (498 ± 90 ms) than for the large distance (523 ± 97 ms).

### Meta-analysis Results

**FIGURE 2:**
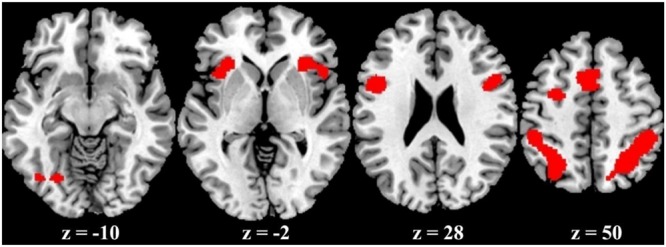
**ALE activation maps from the meta-analysis**.

The ALE meta-analysis revealed a high convergence across independent studies on the brain regions involved in number processing, including bilateral parietal lobes, bilateral precentral gyrus, bilateral insula, left cingulate gyrus, left middle frontal gyrus, and left fusiform gyrus (**Table 1**; **Figure 2**). Since both tasks activated a large portion of bilateral parietal lobe, we then defined ROIs as the spheres with a 3mm radius centering on the peak voxels within the left and right parietal lobes, respectively. These ROIs served as masks for the following fMRI analysis. It should be noted that this study defined ROIs based on the consistent results of the meta-analysis and these ROIs yielded a significant PDE and CDE in the current study (see below). The ROIs defined from the meta-analysis should be more powerful and more accurate than defined by our own data. Therefore, we reported in the main text the results based on the ROIs defined according to the meta-analysis, and included whole brain results in the supplementary Materials. As shown in Supplementary Figure S3, there were few differences between the CDE and PDE outside the parietal ROIs. The only difference was that the CDE, but the PDE, had a significant hemodynamic signature in the right insular ROI.

**Table 1:** Concordant areas for numerical processing based on the meta-analysis.

Hem.	Brain area	BA	*x*	*y*	*z*	ALE	Vol./mm^3^
R	Inferior parietal lobule	40	44	-40	50	0.045	11920
R	Superior parietal lobule	7	28	-58	46	0.039	
R	Inferior parietal lobule	40	36	-44	40	0.039	
R	Precuneus	7	20	-66	54	0.027	
R	Precuneus	7	24	-64	32	0.019	
R	Precuneus	31	20	-64	34	0.019	
L	Inferior parietal lobule	40	-36	-48	42	0.042	11008
L	Superior parietal lobule	7	-28	-62	48	0.041	
L	Inferior parietal lobule	40	-46	-38	46	0.037	
L	Cingulate gyrus	24	-6	12	48	0.028	3848
L	Cingulate gyrus	24	2	4	46	0.027	
L	Cingulate gyrus	32	2	22	38	0.020	
R	Precentralgyrus	6	46	6	30	0.040	3440
L	Precentralgyrus	6	-48	4	28	0.043	2864
R	Insula	13	34	24	0	0.038	2776
R	Precentralgyrus	44	50	16	2	0.023	
L	Insula	13	-30	24	2	0.034	2464
L	Insula	13	-38	16	0	0.028	
L	Middle frontal gyrus	6	-28	-2	54	0.034	1568
L	Fusiform gyrus	19	-26	-70	-8	0.022	440
L	Fusiform gyrus	19	-42	-70	-8	0.018	304

*Coordinates (*x, y, z*) are reported as MNI coordinates; Hem., Hemisphere; L, Left; R, Right; BA, Brodmann area; ALE, Activation likelihood estimate; Vol., volume.*

### Imaging Results

**FIGURE 3:**
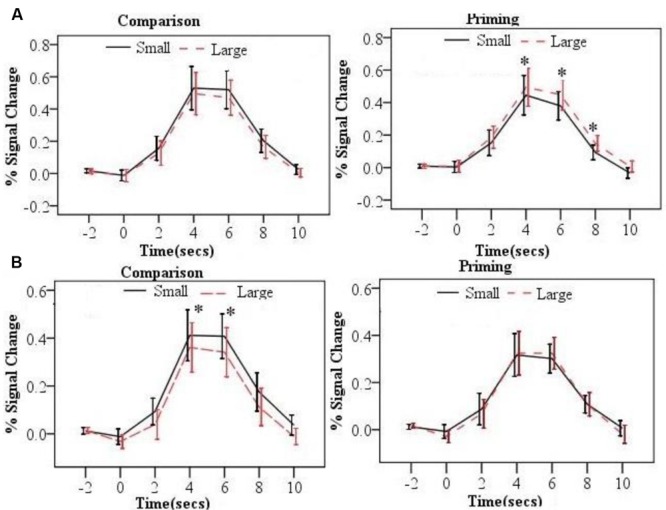
**The time courses by distance (small and large) and task (comparison and priming) for the left parietal lobe ROI **(A)**, the right parietal ROI (B)**. Error bars represent 95% confidence interval (**^∗^***p* < 0.05).

The time course of the BOLD signal for each trial was extracted by the PEATE for each task and each ROI. Across ROIs and tasks, the activation of each trial reached its peak around 6s after the presentation of the second number. We then tested whether the numerical distance in each task was associated with changes in BOLD signal. Specifically, repeated-measure ANOVAs were conducted with task and distance as two within-subject factors in each 2s time points (5 time points in total) for each brain region. Results revealed significant distance × task interactions in the bilateral parietal ROIs. **Table 2** shows all the ANOVAs results in these two ROIs. **Figure 3** shows the time courses of the two distance conditions for the comparison and priming tasks for bilateral parietal ROIs. The time courses of the two distance conditions for the comparison and priming tasks for other ROIs are shown in Supplemental Materials.

**Table 2:** The ANOVA results in five ROIs (Regions of Interests).

		2 s	4 s	6 s	8 s	10 s
Left parietal	D × T	*p* = 0.081	*p* = 0.017	*p* = 0.005	*p* = 0.022	*p* = 0.122
	Simple effects		CDE (*p* = 0.153)	CDE (*p* = 0.123)	CDE (*p* = 0.196)	
			PDE (*p* = 0.050)	PDE (*p* = 0.013)	PDE (*p* = 0.013)	
			STE (*p* = 0.083)	STE (*p* = 0.008)	STE (*p* = 0.002)	
			LTE (*p* = 0.974)	LTE (*p* = 0.692)	LTE (*p* = 0.637)	
Right parietal	D × T	*p* = 0.352	*p* = 0.067	*p* = 0.001	*p* = 0.121	*p* = 0.613
	Simple effects		CDE (*p* = 0.018)	CDE (*p* = 0.007)		
			PDE (*p* = 0.748)	PDE (*p* = 0.397)		
			STE (*p* = 0.039)	STE (*p* = 0.015)		
			LTE (*p* = 0.349)	LTE (*p* = 0.727)		

*D, distance; T, task; STE, the task effect with the small distance; LTE, the task effect with the large distance.*

As listed in the **Table 2**, the neural PDE (large > small distance in neural activation) was observed in the left parietal lobe. In contrast, the neural CDE (small > large distance in neural activation) was observed in the right parietal lobe. In order to confirm whether there was a hemispheric laterality for the PDE and CDE, repeated-measure ANOVAs were conducted with hemisphere (left/right) and distance (small/large) as two within-subject factors per task. Results showed significant interactions between hemisphere and distance at 4, 6, and 8 s time points for the priming task (*F*s > 5.903, *p*s < 0.049). Further analyses showed that the PDE was significant on the left hemisphere (*p*s < 0.050) but not on the right hemisphere (*p*s > 0.397). However, only the distance main effect was observed at 4s and 6s time points for the comparison task (*F*s > 4.703, *p*s < 0.041) and no significant interaction between hemisphere and distance was found (*p*s > 0.353). These findings revealed hemispheric laterality for the PDE but not for the CDE.

## Discussion

Using fMRI with a slow event-related design, the present study aimed to examine whether the CDE and PDE shared the same neural mechanism. Our fMRI results revealed robust neural CDE and PDE. The CDE had a significant hemodynamic signature in the right parietal ROI but not in the left parietal ROI, although a direct test of this hemispheric laterality did not reach statistical significance. In contrast, the PDE showed significant left hemispheric laterality with a significant hemodynamic signature in the left parietal ROI but not in the right parietal ROI. These results led us to conclude that the CDE and PDE may have different underlying neural mechanisms.

Our finding that the CDE had a significant hemodynamic signature in the right parietal cortex but not in the left parietal cortex is in line with some previous studies (Dehaene, 1996; Andres et al., 2005; Cohen Kadosh et al., 2012). For example, Dehaene (1996) found a right-lateralized parietal dominance for the CDE in an event-related potentials (ERPs) study. This finding is also compatible with the finding by Andres et al. (2005). Using a symbolic number comparison task, Andres et al. (2005) found a significant increase in RTs when comparing digits close to 5 after a disruption of either the left posterior parietal cortex (PPC) alone or bilateral PPC simultaneously. However, the comparison of digits far from 5 was unaltered by disrupting only one PPC but RTs increased after bilateral PPC stimulation. They concluded that processing the small-distance trials mainly relied on the left parietal cortex, but processing the large distance trials involved both the left and right parietal cortex, suggesting that the neural CDE should be mainly associated with the right parietal cortex.

The second important and novel finding of this study was that the PDE showed a significant hemodynamic signature in the left parietal cortex. To our knowledge, there are only two neuroimaging studies of the PDE (Notebaert et al., 2010; Sasanguie et al., 2013). However, unlike this study, both involved cross-notation priming. One study (Notebaert et al., 2010) investigated the cross-notation priming effect (Arabic and verbal numerals) and revealed a bilateral parietal activation when an Arabic numeral preceded a verbal numeral. The other study disrupted processing in the left and right parietal regions with repetitive transcranial magnetic stimulation (rTMS) during the symbolic and non-symbolic priming tasks (Sasanguie et al., 2013). Results showed that the PDE between symbolic and non-symbolic numerosities was disrupted by the left parietal TMS, suggesting a crucial role of the left hemisphere for the mapping between small symbolic and non-symbolic numerosities.

Taken together, our study found that the CDE had a significant hemodynamic signature in the right parietal cortex and the PDE in the left parietal cortex. One possible explanation for this finding is that the right parietal cortex is superior for spatial processing of magnitude while the left parietal cortex is superior for semantic processing of magnitude. Specifically, spatial processing of magnitude might be linked to some visuo-spatial processes when attending to specific quantities on the number line. Such number-based attention is particularly needed in the comparison task where subjects have to decide which of two quantities is the larger (Pesenti et al., 2000; Pinel et al., 2001; Dehaene et al., 2003). The small distance in the comparison task required more number-based attention and discrimination than the large distance. In contrast, semantic processing of magnitude might be linked to semantic representation of the quantity that the numbers represent. In this study, the small prime distance (e.g., prime 4 and target 2) required less numerical semantic representations (only 3 was primed and represented) than the large prime distance (e.g., 5, 4, and 3 were primed and represented for prime 6 and target 2). Another alternative explanation for the hemispheric differences between the CDE and PDE is that the comparison task required response selection whereas the priming task did not. Response selection during number comparison could lead to right parietal activation (Gobel et al., 2004). However, both our explanations are hypothetical and we tend to leave open what the exact cause is of the hemispheric differences between priming and comparison.

Finally, one particular limitation of our experimental design needs to be discussed. As stated above, the comparison task required response selection whereas the priming task did not. As shown by Gobel et al. (2004), response selection during number comparison can lead to both the left and right parietal activation. As a result, differences between small- and large-distance trials in the comparison task in this study might be due to increasing response selection difficulty in small-distance trials compared to large-distance trials. In future studies, a more optimal design can be adopted to compare the PDE and CDE in the same task as done previously (e.g., Naccache and Dehaene, 2001).

## Conclusion

We used a meta-analysis and two independent but matched tasks to investigate the neural mechanisms for the CDE and PDE. Our results provide the first imaging evidence that the CDE and PDE have different underlying mechanisms, with the CDE having a significant hemodynamic signature in the right parietal cortex and the PDE in the left parietal cortex.

## Author Contributions

Conceived and designed the experiments: LZ and FC. Performed the experiments: FC. Analyzed the data: FC, QH, CC. Wrote the paper: LZ, QH, and CC.

## Conflict of Interest Statement

The authors declare that the research was conducted in the absence of any commercial or financial relationships that could be construed as a potential conflict of interest.

## Footnotes

1 http://www.brainmap.org/ale/

2 http://www.fmrib.ox.ac.uk/fsl

3 http://www.jonaskaplan.com/peate

## References

[B1] Andres M. Seron X. Olivier E. (2005). Hemispheric lateralization of number comparison. *Cogn. Brain Res.* 251 283–290. 10.1016/j.cogbrainres.2005.06.002 16005617

[B2] Arsalidou M. Taylor M. J. (2011). Is 2+ 2 = 4? Meta-analyses of brain areas needed for numbers and calculations. *Neuroimage* 543 2382–2393. 10.1016/j.neuroimage.2010.10.009 20946958

[B3] Cantlon J. F. Brannon E. M. Carter E. J. Pelphrey K. A. (2006). Functional imaging of numerical processing in adults and 4-y-old children. *PLoS Biol.* 4:e125 10.1371/journal.pbio.0040125 PMC1431577 16594732

[B4] Cohen Kadosh R. Bien N. Sack A. T. (2012). Automatic and intentional number processing both rely on intact right parietal cortex: a combined FMRI and neuronavigated TMS study. *Front. Hum. Neurosci.* 6:2 10.3389/fnhum.2012.00002 PMC3269809 22347175

[B5] De Smedt B. Verschaffel L. Ghesquière P. (2009). The predictive value of numerical magnitude comparison for individual differences in mathematics achievement. *J. Exp. Child Psychol.* 103 469–479. 10.1016/j.jecp.2009.01.010 19285682

[B6] Defever E. Sasanguie D. Vanderwaetere M. Reynvoet B. (2012). What can the same-different task tell us about the development of magnitude representations? *Acta Psychol.* 140 35–42. 10.1016/j.actpsy.2012.02.005 22426429

[B7] Dehaene S. (1996). The organization of brain activations in number comparison: event-related potentials and the additive-factors method. *J. Cogn. Neurosci.* 8 47–68. 10.1162/jocn.1996.8.1.47 23972235

[B8] Dehaene S. (1997). *The Number Sense: How the Mind Creates Mathematics*. New York, NY: Oxford University Press.

[B9] Dehaene S. Piazza M. Pinel P. Cohen L. (2003). Three parietal circuits for number processing. *Cogn. Neuropsychol.* 20 487–506. 10.1080/02643290244000239 20957581

[B10] Dormal V. Pesenti M. (2009). Common and specific contributions of the intraparietal sulci to numerosity and length processing. *Hum. Brain Mapp.* 30 2466–2476. 10.1002/hbm.20677 19294652 PMC6870899

[B11] Eger E. Sterzer P. Russ M. O. Giraud A. L. Kleinschmidt A. (2003). A supramodal number representation in human intraparietal cortex. *Neuron* 37 719–726. 10.1016/S0896-6273(03)00036-9 12597867

[B12] Eickhoff S. B. Bzdok D. Laird A. R. Kurth F. Fox P. T. (2012). Activation likelihood estimation meta-analysis revisited. *Neuroimage* 593 2349–2361. 10.1016/j.neuroimage.2011.09.017 PMC3254820 21963913

[B13] Eickhoff S. B. Laird A. R. Grefkes C. Wang L. E. Zilles K. Fox P. T. (2009). Coordinate-based activation likelihood estimation meta-analysis of neuroimaging data: a random-effects approach based on empirical estimates of spatial uncertainty. *Hum. Brain Mapp.* 309 2907–2926. 10.1002/hbm.20718 PMC2872071 19172646

[B14] Fias W. Lammertyn J. Reynvoet B. Dupont P. Orban G. A. (2003). Parietal representation of symbolic and nonsymbolic magnitude. *J. Cogn. Neurosci.* 15 47–56. 10.1162/089892903321107819 12590842

[B15] Gobel S. M. Johansen-Berg H. Behrens T. E. Rushworth M. F. S. (2004). Response-selection-related parietal activation during number comparison. *J. Cogn. Neurosci.* 16 1536–1551. 10.1162/0898929042568442 15601517

[B16] Holloway I. D. Ansari D. (2009). Mapping numerical magnitudes onto symbols: the numerical distance effect and individual differences in children’s mathematics achievement. *J. Exp. Child Psychol.* 103 17–29. 10.1016/j.jecp.2008.04.001 18513738

[B17] Jenkinson M. Smith S. (2001). A global optimisation method for robust affine registration of brain images. *Med. Image Anal.* 52 143–156. 10.1016/S1361-8415(01)00036-6 11516708

[B18] Kaufmann L. Wood G. Rubinsten O. Henik A. (2011). Meta-analyses of developmental fMRI studies investigating typical and atypical trajectories of number processing and calculation. *Dev. Neuropsychol.* 366 763–787. 10.1080/87565641.2010.549884 21761997

[B19] Laird A. R. Fox P. M. Price C. J. Glahn D. C. Uecker A. M. Lancaster J. L. (2005). ALE meta-analysis, controlling the false discovery rate and performing statistical contrasts. *Hum. Brain Mapp.* 251 155–164. 10.1002/hbm.20136 PMC6871747 15846811

[B20] Moyer R. S. Landauer T. K. (1967). Time required for judgments of numerical inequality. *Nature* 215 1519–1520. 10.1038/2151519a0 6052760

[B21] Naccache L. Dehaene S. (2001). Unconscious semantic priming extends to novel unseen stimuli. *Cognition* 80 215–229. 10.1016/S0010-0277(00)00139-6 11274983

[B22] Nieder A. Dehaene S. (2009). Representation of number in the brain. *Annu. Rev. Neurosci.* 32 185–208. 10.1146/annurev.neuro.051508.135550 19400715

[B23] Notebaert K. Pesenti M. Reynvoet B. (2010). The neural origin of the priming distance effect, distance-dependent recovery of parietal activation using symbolic magnitudes. *Hum. Brain Mapp.* 315 669–677. 10.1002/hbm.20896 PMC6870915 19882648

[B24] Pesenti M. Thioux M. Seron X. De Volder A. (2000). Neuroanatomical substrates of Arabic number processing, numerical comparison, and simple addition, A PET study. *J. Cogn. Neurosci.* 12 461–479. 10.1162/089892900562273 10931772

[B25] Piazza M. Pinel P. Le Bihan D. Dehaene S. (2007). A magnitude code common to numerosities and number symbols in human intraparietal cortex. *Neuron* 53 293–305. 10.1016/j.neuron.2006.11.022 17224409

[B26] Pinel P. Dehaene S. Riviere D. LeBihan D. (2001). Modulation of parietal activation by semantic distance in a number comparison task. *Neuroimage* 14 1013–1026. 10.1006/nimg.2001.0913 11697933

[B27] Restle F. (1970). Speed of adding and comparing numbers. *J. Exp. Psychol.* 83 274–278. 10.1186/1471-2334-11-269

[B28] Reynvoet B. Brysbaert M. Fias W. (2002). Semantic priming in number naming. *Q. J. Exp. Psychol. A.* 554 1127–1139. 10.1080/02724980244000116 12420988

[B29] Reynvoet B. De Smedt B. Van den Bussche E. (2009). Children’s representation of symbolic magnitude: the development of the priming distance effect. *J. Exp. Child Psychol.* 103 480–489. 10.1016/j.jecp.2009.01.007 19285684

[B30] Sasanguie D. Defever E. van den Bussche E. Reynvoet B. (2011). The reliability of and the relation between non-symbolic numerical distance effects in comparison same-different judgments and priming. *Acta Psychol.* 1361 73–80. 10.1016/j.actpsy.2010.10.004 21075357

[B31] Sasanguie D. Göbel S. M. Reynvoet B. (2013). Left parietal TMS disturbs priming between symbolic and non-symbolic number representations. *Neuropsychologia* 51 1528–1533. 10.1016/j.neuropsychologia.2013.05.001 23665379

[B32] Sekuler R. Mierkiewicz D. (1977). Children’s judgments of numerical inequality. *Child Dev.* 48 630–633. 10.2307/1128664

[B33] Sescousse G. Caldú X. Segura B. Dreher J. C. (2013). Processing of primary and secondary rewards, a quantitative meta-analysis and review of human functional neuroimaging studies. *Neurosci. Biobehav. Rev.* 374 681–696. 10.1016/j.neubiorev.2013.02.002 23415703

[B34] Turkeltaub P. E. Eickhoff S. B. Laird A. R. Fox M. Wiener M. Fox P. (2012). Minimizing within-experiment and within-group effects in activation likelihood estimation meta-analyses. *Hum. Brain Mapp.* 331 1–13. 10.1002/hbm.21186 PMC4791073 21305667

[B35] Van Opstal F. Gevers W. De Moor W. Verguts T. (2008). Dissecting the symbolic distance effect: comparison and priming effects in numerical and nonnumerical orders. *Psychon. Bull. Rev.* 152 419–425. 10.3758/PBR.15.2.419 18488662

