# Supplementary material for: Different Neural Mechanisms for the Comparison and Priming Distance Effects: An fMRI Study
Source: Front Psychol. 2016 Oct 26;7:1633. doi: 10.3389/fpsyg.2016.01633 (PMC5080342; doi:10.3389/fpsyg.2016.01633)
Supplement: Supplementary file 1 [file Data_Sheet_1.DOCX]

**Supplemental Materials**

Table S1 Descriptive information of studies and contrasts included for the meta-analysis

| Authors | Year | Sample | | | | Included contrasts |
| --- | --- | --- | --- | --- | --- | --- |
|  |  | No. | F | Hand | Age |  |
| Dehaene et al. | 1999 | 7 | 4 | R | 25 | Approximate > exact arithmetic |
| Pinel et al. | 1999 | 11 | 2 | R | 25.7 | Numerical comparison distance effect: small > large |
| Le Clec’H et al. | 2000 | 6 | 3 | NA | 27 | Numerals > body parts |
| Gruber et al | 2001 | 6 | 0 | R | 25.8 | ^b^Compound calculation > baseline |
| Pinel et al. | 2001 | 26 | 10 | R | 25 | Numerical comparison distance effect: small > large |
| Thioux et al. | 2001 | 6 | 0 | NA | NA | Numerical semantic tasks > animal names semantic task |
| Eger et al. | 2003 | 9 | 5 | R | 27.9 | Numbers identification > letters identification |
| Gobel et al. | 2004 | 12 | 6 | R | 26.7 | Numerical comparison > rest |
| Thompson et al. | 2004 | 12 | 6 | R | 34^a^ | Viewing finger-representing numbers > control |
| Ansari et al. | 2005 | 12 | NA | NA | 19.8 | Numerical comparison distance effect: small > large |
| Kadosh et al. | 2005 | 15 | 8 | 12R | 27.8 | Numerical comparison > size comparison |
| Kaufmann et al. | 2005 | 17 | 7 | R | 31.1 | Numerical comparison > null events |
| Venkatraman et al. | 2005 | 10 | 3 | R | 22.5^a^ | Symbolic approximate addition > control |
| Ansari and Dhital | 2006 | 9 | 3 | R | 19.8 | Numerical comparison distance effect |
| Ansari et al. | 2006 | 14 | 8 | R | 21.3 | Numerical comparison distance effect: small > large |
| Cantlon et al. | 2006 | 12 | 5 | NA | 25 | Numerical deviants > shape deviants |
| Liu et al. | 2006 | 12 | 7 | R | 31.5^a^ | Numerical comparison > baseline |
| Tang et al. | 2006 | 18 | 7 | R | 25 | Numerical distance > physical distance |
| Zhou et al. | 2006 | 12 | 6 | R | 21.4 | Numerical sequences forward |
| Fehr et al. | 2007 | 11 | 6 | R | 26.8 | Subtraction: ^b^complex> ^c^simple |
| Fias et al | 2007 | 17 | 9 | 13R | 28.5^a^ | Numerical comparison vs control > letter comparison vs control |
| Ischebeck et al. | 2007 | 18 | 9 | R | 27.8 | Multiplication: ^d^novel > ^e^repeated |
| Masataka et al. | 2007 | 14 | 9 | R | 24.5^a^ | Learning to read Roman numerals |
| Piazza et al. | 2007 | 14 | NA | R | NA | Deviations in magnitude: far > close distance |
| Kaufmann et al. | 2008 | 12 | 6 | R | 33.2 | Numerical comparison > rest |
| Wood et al. | 2008 | 17 | 0 | R | 24.2 | Number bisection: small > large distance |
| Zago et al | 2008 | 14 | 8 | R | 23.5^a^ | Numbers manipulation > maintenance |
| Chiao et al. | 2009 | 12 | 6 | NA | 20.7 | Symbolic numerical comparison > baseline |
| Libertus et al. | 2009 | 15 | 8 | NA | 25 | Two-back tasks: digits > letters and faces |
| Troiani et al. | 2009 | 14 | NA | NA | 24.4 | Numerical quantifiers > logical quantifiers |
| Dormal & Pesenti | 2009 | 14 | 0 | R | 21 | Numerosity comparison > rest |
| Ischebeck et al. | 2009 | 17 | 7 | NA | 25 | Multiplication: ^f^untrained > ^g^trained |
| Klein et al. | 2009 | 19 | 0 | R | 23.6 | Addition: ^h^small > ^i^large result-distractor distance |
| Dormal et al. | 2010 | 15 | 0 | R | 21 | Simultaneous numerosity > fixtation |
| Holloway et al. | 2010 | 19 | 10 | R | 23.5 | Conjunction：symbolic & nonsymbolic comparison |
| Klein et al. | 2010a | 17 | 0 | R | 24.9 | Numerical comparison > baseline |
| Klein et al. | 2010b | 17 | 0 | R | 28 | Multi-digit addition: activation varying with problem size |
| Notebaert et al. | 2010a | 13 | 0 | R | 24.5^a^ | Numerical adaptation > null events |
| Notebaert et al. | 2010b | 13 | 6 | R | 25.5^a^ | Deviations in magnitude: far > close |

Table S1 *(Continued)*

| Authors | Year | Sample | | | | Selected contrasts |
| --- | --- | --- | --- | --- | --- | --- |
|  |  | No. | F | Hand | Age |  |
| Prado et al. | 2010 | 15 | 12 | R | 23 | Numerical comparison distance effect: small > large |
| ^j^Santens et al.j | 2010 | 16 | 3 | 15R | 22.2 | Conjunction activation increasing with magnitudes |
|  |  | 12 | 0 | R | 19.9 | Activation increasing with magnitudes in dots |
| Gullick et al. | 2011a | 17 | 8 | R | 21.4 | Numerical comparison distance effect: close > far |
| Gullick & Temple | 2011b | 32 | 16 | R | 20.2 | Numbers > dates |
| Kadosh et al. | 2011 | 19 | 12 | NA | 26.3 | Deviations in magnitude for digits |
| Price & Ansari | 2011 | 19 | 6 | R | 22.2 | Viewing digits > viewing letters and unfamiliar symbols |
| Roggeman et al. | 2011 | 23 | 0 | R | 25.8 | Deviations in magnitude: large > small |
| Gullick et al. | 2012 | 24 | 12 | R | 20.3 | Positive comparison distance effect: small > large |
| Kallai et al. | 2012 | 40 | 20 | NA | 20.9 | Deviations in magnitude: far > close |
| Tschentscher et al. | 2012 | 29 | 15 | R | 25 | Numbers > baseline |
| Zhang et al. | 2012 | 20 | 10 | R | 20.6 | Numerical semantic tasks > baseline |
| Eger et al. | 2013 | 14 | 6 | NA | 23.7 | Numerical comparison distance effect |
| Holloway et al. | 2013 | 26 | 8 | NA | 25 | Numerical comparison distance effect |
| Kim et al. | 2013 | 24 | 14 | NA | 30^a^ | Numerical comparison distance effect |
| Vogel et al. | 2013 | 14 | 7 | R | 24.6 | Number line estimation > control |
| Attout et al. | 2014 | 26 | 15 | R | NA | Numerical order judgment |
| Bulthe et al. | 2014 | 16 | 12 | 14R | 24.5^a^ | Subtraction > fixation |
| Demeyere et al. | 2014 | 12 | 9 | 10R | 26 | Repeat numerosities: large > small |
| Wei et al. | 2014 | 20 | 10 | R | 20.6 | Symbolic numerical semantic tasks > baseline |

No., number of participants; F, Female; R, right; #Con., number of reported contrasts; NA, not reported.

a Middle value of age range.

b Problems with large operands.

c Problems with small operands.

d Novel multiplication refers to multiplication problems which were repeated 3 times.

e Repeated multiplication refers to multiplication problems which were repeated 24 times.

f Untrained multiplication refers to multiplication problems on which participants did not receive training.

g Trained multiplication refers to multiplication problems on which participants did receive training.

h Alternative solutions with small distracter distance from correct solution.

i Alternative solutions with large distracter distance from correct solution.

j This study includes two independent experiments.


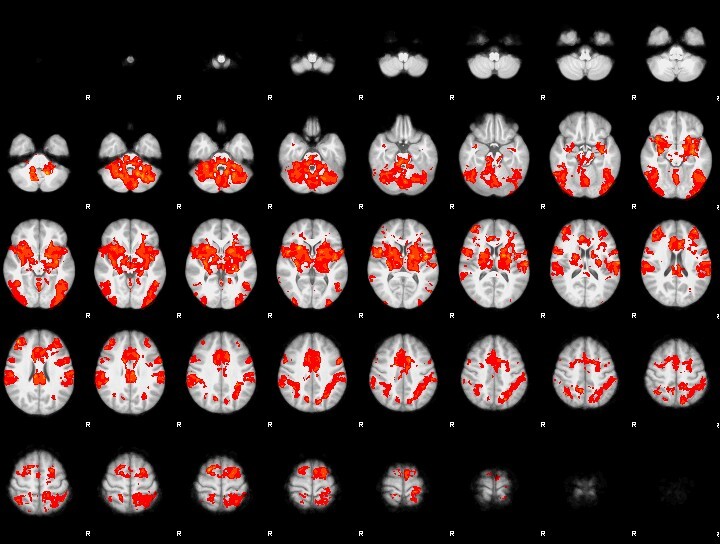


Figure S1. The brain activation maps for the comparison task based on our fMRI data


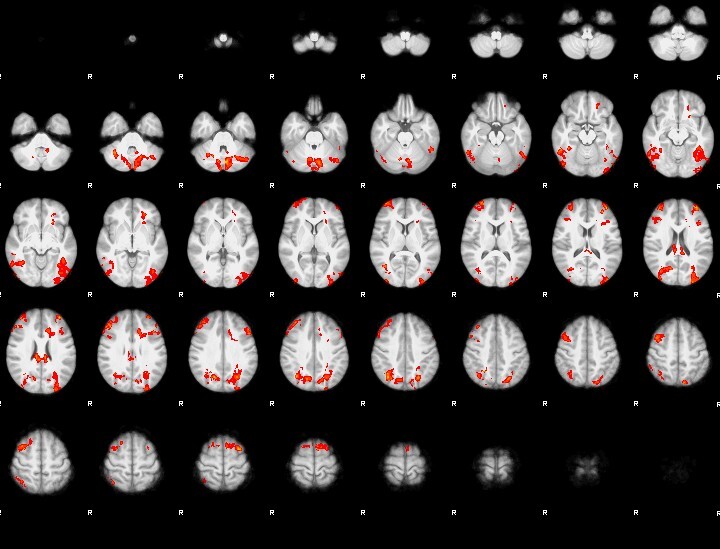
Figure S2. The brain activation maps for the priming task based on our fMRI data


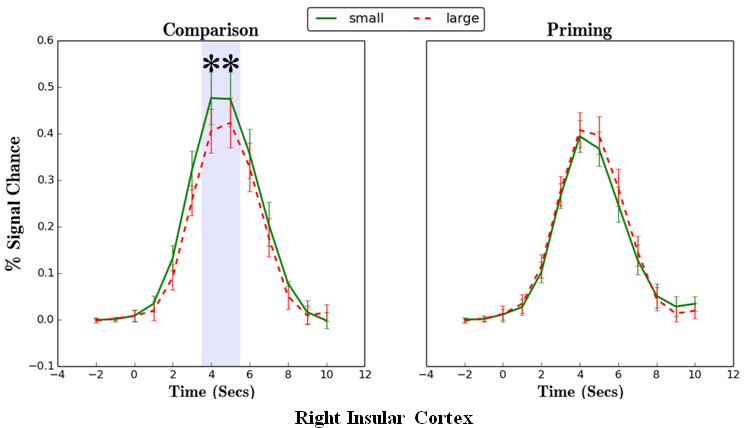


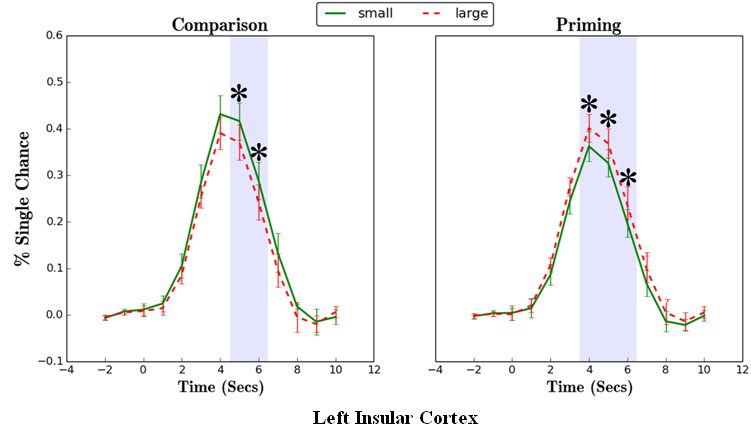


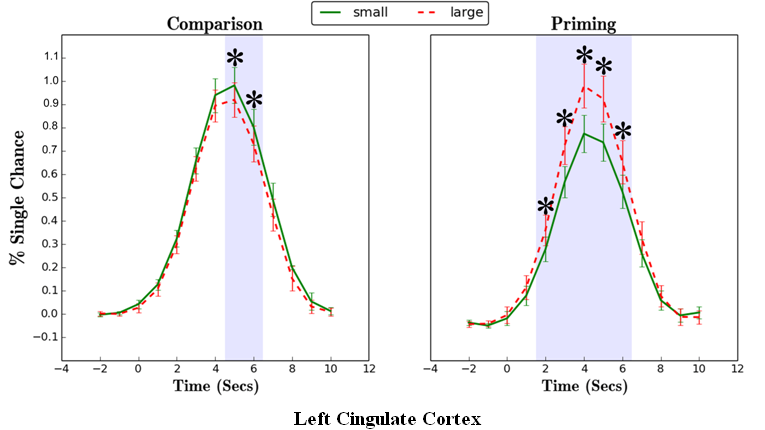


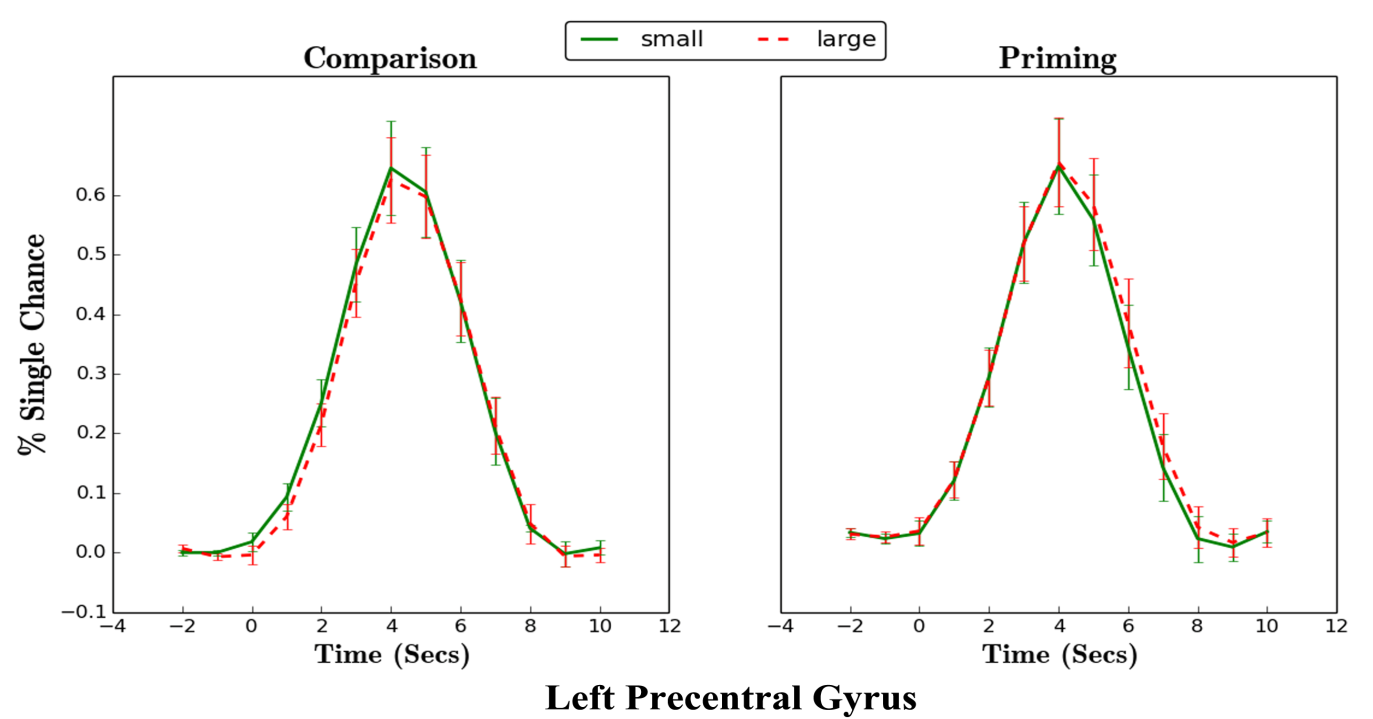


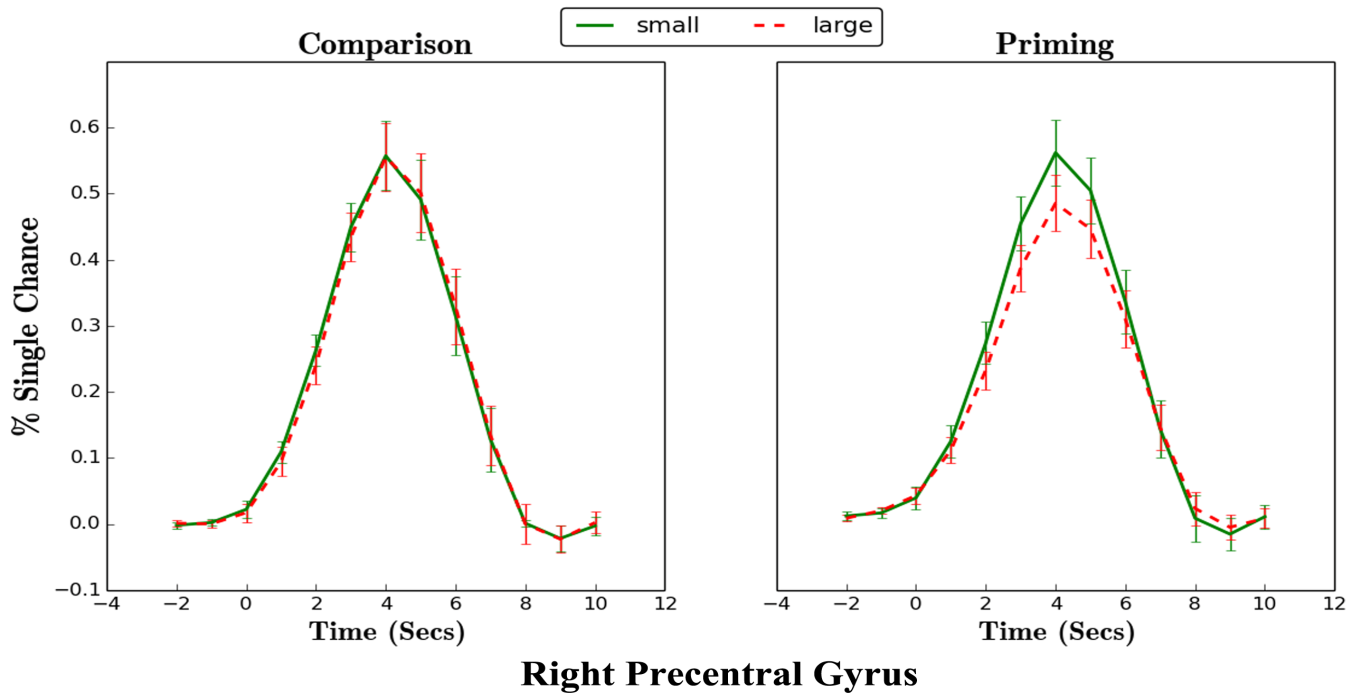

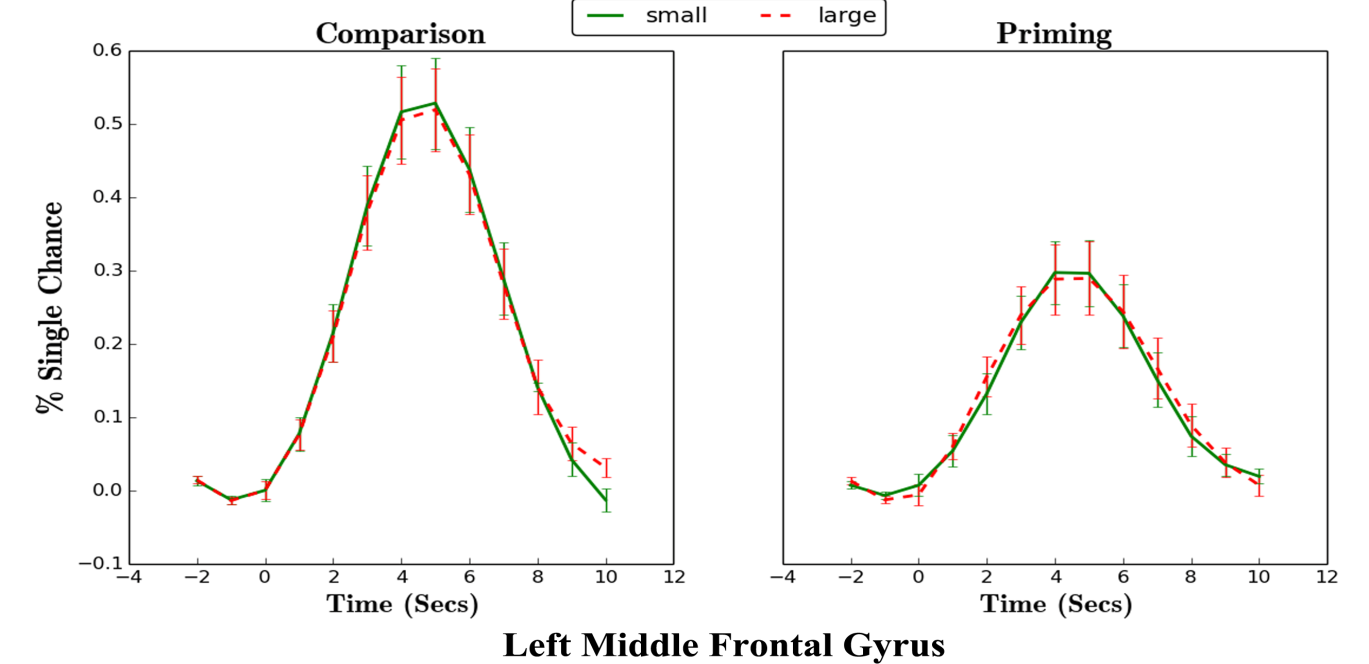

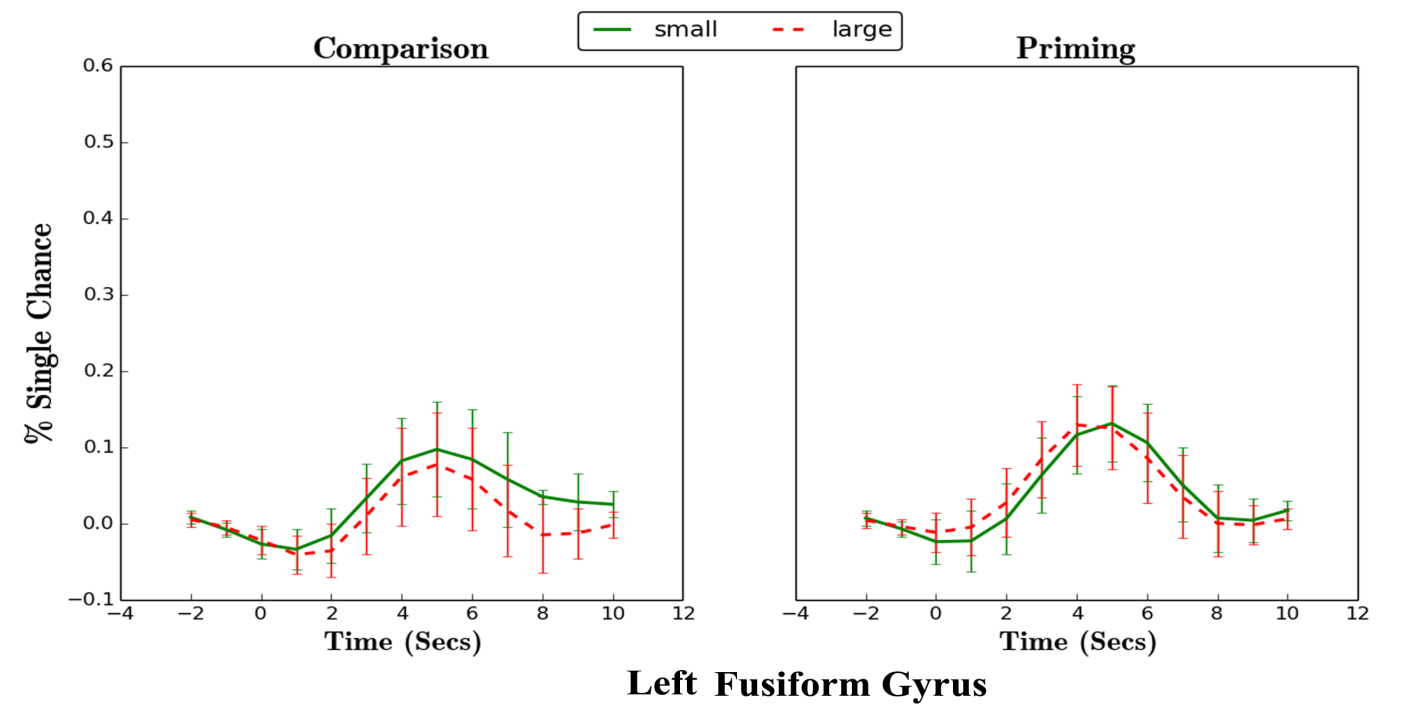


Figure S3. The time courses of the two distance conditions are shown for bilateral insular ROIs, left cingulate ROI, bilateral precentral ROIs, left middle frontal ROI and left fusiform ROI. Error bars represent the standard errors. ***** *p* < .05.

**References**

Ansari, D., & Dhital, B. (2006). Age-related changes in the activation of the intraparietal sulcus during nonsymbolic magnitude processing: an event-related functional magnetic resonance imaging study. *Journal of Cognitive Neuroscience*, *18*(11), 1820-1828.

Ansari, D., Fugelsang, J. A., Dhital, B., & Venkatraman, V. (2006). Dissociating response conflict from numerical magnitude processing in the brain: an event-related fMRI study. *NeuroImage*, *32*(2), 799-805.

Ansari, D., Garcia, N., Lucas, E., Hamon, K., & Dhital, B. (2005). Neural correlates of symbolic number processing in children and adults. *Neuroreport*, *16*(16), 1769-1773.

Attout, L., Fias, W., Salmon, E., & Majerus, S. (2014). Common Neural Substrates for Ordinal Representation in Short-Term Memory, Numerical and Alphabetical Cognition. *PloS one*, *9*(3), e92049.

Bulthé, J., De Smedt, B., & Op de Beeck, H. P. (2014). Format-dependent representations of symbolic and non-symbolic numbers in the human cortex as revealed by multi-voxel pattern analyses. *NeuroImage*, *87*, 311-322.

Cantlon, J. F., Brannon, E. M., Carter, E. J., & Pelphrey, K. A. (2006). Functional imaging of numerical processing in adults and 4-y-old children. *PLoS biology*, *4*(5), e125.

Chiao, J. Y., Harada, T., Oby, E. R., Li, Z., Parrish, T., & Bridge, D. J. (2009). Neural representations of social status hierarchy in human inferior parietal cortex. *Neuropsychologia*, *47*(2), 354-363.

Dehaene, S., Spelke, E., Pinel, P., Stanescu, R., & Tsivkin, S. (1999). Sources of mathematical thinking: Behavioral and brain-imaging evidence. *Science*, *284*(5416), 970-974.

Demeyere, N., Rotshtein, P., & Humphreys, G. W. (2014). Common and dissociated mechanisms for estimating large and small dot arrays: Value‐specific fMRI adaptation. *Human brain mapping*.

Dormal, V., & Pesenti, M. (2009). Common and specific contributions of the intraparietal sulci to numerosity and length processing. *Human brain mapping*, *30*(8), 2466-2476.

Dormal, V., Andres, M., Dormal, G., & Pesenti, M. (2010). Mode-dependent and mode-independent representations of numerosity in the right intraparietal sulcus. *Neuroimage*, *52*(4), 1677-1686.

Eger, E., Pinel, P., Dehaene, S., & Kleinschmidt, A. (2013). Spatially Invariant Coding of Numerical Information in Functionally Defined Subregions of Human Parietal Cortex. *Cerebral Cortex*, bht323.

Eger, E., Sterzer, P., Russ, M. O., Giraud, A. L., & Kleinschmidt, A. (2003). A supramodal number representation in human intraparietal cortex. *Neuron*, *37*(4), 719-726.

Fehr, T., Code, C., & Herrmann, M. (2007). Common brain regions underlying different arithmetic operations as revealed by conjunct fMRI–BOLD activation. *Brain research*, *1172*, 93-102.

Fias, W., Lammertyn, J., Caessens, B., & Orban, G. A. (2007). Processing of abstract ordinal knowledge in the horizontal segment of the intraparietal sulcus. *The Journal of Neuroscience*, *27*(33), 8952-8956.

Göbel, S. M., Johansen-Berg, H., Behrens, T., & Rushworth, M. F. (2004). Response-selection-related parietal activation during number comparison. *Journal of Cognitive Neuroscience*, *16*(9), 1536-1551.

Gruber, O., Indefrey, P., Steinmetz, H., & Kleinschmidt, A. (2001). Dissociating neural correlates of cognitive components in mental calculation. *Cerebral Cortex*, *11*(4), 350-359.

Gullick, M. M., & Temple, E. (2011b). Are historic years understood as numbers or events? An fMRI study of numbers with semantic associations. *Brain and cognition*, *77*(3), 356-364.

Gullick, M. M., Wolford, G., & Temple, E. (2012). Understanding less than nothing: neural distance effects for negative numbers. *Neuroimage*, *62*(1), 542-554.

Holloway, I. D., Battista, C., Vogel, S. E., & Ansari, D. (2013). Semantic and perceptual processing of number symbols: Evidence from a cross-linguistic fmri adaptation study. *Journal of cognitive neuroscience*, *25*(3), 388-400.

Holloway, I. D., Price, G. R., & Ansari, D. (2010). Common and segregated neural pathways for the processing of symbolic and nonsymbolic numerical magnitude: an fMRI study. *Neuroimage*, *49*(1), 1006-1017.

Ischebeck, A., Zamarian, L., Egger, K., Schocke, M., & Delazer, M. (2007). Imaging early practice effects in arithmetic. *Neuroimage*, *36*(3), 993-1003.

Ischebeck, A., Zamarian, L., Schocke, M., & Delazer, M. (2009). Flexible transfer of knowledge in mental arithmetic—An fMRI study. *Neuroimage*, *44*(3), 1103-1112.

Kadosh, R. C., Bahrami, B., Walsh, V., Butterworth, B., Popescu, T., & Price, C. J. (2011). Specialization in the human brain: the case of numbers. *Frontiers in human neuroscience*, *5*.

Kadosh, R. C., Henik, A., Rubinsten, O., Mohr, H., Dori, H., van de Ven, V., ... & Linden, D. E. (2005). Are numbers special?: the comparison systems of the human brain investigated by fMRI. *Neuropsychologia*, *43*(9), 1238-1248.

Kallai, A. Y., Schunn, C. D., & Fiez, J. A. (2012). Mental arithmetic activates analogic representations of internally generated sums. *Neuropsychologia*, *50*(10), 2397-2407.

Kaufmann, L., Koppelstaetter, F., Delazer, M., Siedentopf, C., Rhomberg, P., Golaszewski, S., ... & Ischebeck, A. (2005). Neural correlates of distance and congruity effects in a numerical Stroop task: an event-related fMRI study. *Neuroimage*, *25*(3), 888-898.

Kaufmann, L., Vogel, S. E., Wood, G., Kremser, C., Schocke, M., Zimmerhackl, L. B., & Koten, J. W. (2008). A developmental fMRI study of nonsymbolic numerical and spatial processing. *Cortex*, *44*(4), 376-385.

Kim, S. Y., Hashimoto, R. I., Tassone, F., Simon, T. J., & Rivera, S. M. (2013). Altered neural activity of magnitude estimation processing in adults with the fragile X premutation. *Journal of psychiatric research*, *47*(12), 1909-1916.

Klein, E., Moeller, K., Nuerk, H. C., & Willmes, K. (2010a). Research On the neuro-cognitive foundations of basic auditory number processing: an fMRI study.

Klein, E., Nuerk, H. C., Wood, G., Knops, A., & Willmes, K. (2009). The exact vs. approximate distinction in numerical cognition may not be exact, but only approximate: How different processes work together in multi-digit addition. *Brain and cognition*, *69*(2), 369-381.

Klein, E., Willmes, K., Dressel, K., Domahs, F., Wood, G., Nuerk, H. C., & Moeller, K. (2010b). Categorical and continuous-disentangling the neural correlates of the carry effect in multi-digit addition. *Behavioral and Brain Functions*, *6*(1), 70.

Le Clec'H, G., Dehaene, S., Cohen, L., Mehler, J., Dupoux, E., Poline, J. B., ... & Le Bihan, D. (2000). Distinct cortical areas for names of numbers and body parts independent of language and input modality. *Neuroimage*, *12*(4), 381-391.

Libertus, M. E., Brannon, E. M., & Pelphrey, K. A. (2009). Developmental changes in category-specific brain responses to numbers and letters in a working memory task. *Neuroimage*, *44*(4), 1404-1414.

Liu, X., Wang, H., Corbly, C. R., Zhang, J., & Joseph, J. E. (2006). The involvement of the inferior parietal cortex in the numerical Stroop effect and the distance effect in a two-digit number comparison task. *Journal of Cognitive Neuroscience*, *18*(9), 1518-1530.

Margaret M. Gullick, Lisa A. Sprute, Elise Temple (2011a). Individual differences in working memory, nonverbal IQ, and mathematics achievement and brain mechanisms associated with symbolic and nonsymbolic number processing. *Learning and Individual Differ*ences, 21(6), 644-654.

Masataka, N., Ohnishi, T., Imabayashi, E., Hirakata, M., & Matsuda, H. (2007). Neural correlates for learning to read Roman numerals. *Brain and language*, *100*(3), 276-282.

Notebaert, K., Nelis, S., & Reynvoet, B. (2011b). The magnitude representation of small and large symbolic numbers in the left and right hemisphere: an event-related fMRI study. *Journal of cognitive neuroscience*, *23*(3), 622-630.

Notebaert, K., Pesenti, M., & Reynvoet, B. (2010a). The neural origin of the priming distance effect: Distance‐dependent recovery of parietal activation using symbolic magnitudes. *Human brain mapping*, *31*(5), 669-677.

Piazza, M., Pinel, P., Le Bihan, D., & Dehaene, S. (2007). A magnitude code common to numerosities and number symbols in human intraparietal cortex. *Neuron*, *53*(2), 293-305.

Pinel, P., Dehaene, S., Riviere, D., & LeBihan, D. (2001). Modulation of parietal activation by semantic distance in a number comparison task. *Neuroimage*, *14*(5), 1013-1026.

Pinel, P., Le Clec'H, G., van de Moortele, P. F., Naccache, L., Le Bihan, D., & Dehaene, S. (1999). Event-related fMRI analysis of the cerebral circuit for number comparison. *NeuroReport*, *10*(7), 1473-1479.

Prado, J., Noveck, I. A., & Van Der Henst, J. B. (2010). Overlapping and distinct neural representations of numbers and verbal transitive series. *Cerebral Cortex*, *20*(3), 720-729.

Price, G. R., & Ansari, D. (2011). Symbol processing in the left angular gyrus: evidence from passive perception of digits. *Neuroimage*, *57*(3), 1205-1211.

Roggeman, C., Santens, S., Fias, W., & Verguts, T. (2011). Stages of nonsymbolic number processing in occipitoparietal cortex disentangled by fMRI adaptation. *The Journal of Neuroscience*, *31*(19), 7168-7173.

Santens, S., Roggeman, C., Fias, W., & Verguts, T. (2010). Number processing pathways in human parietal cortex. *Cerebral Cortex*, *20*(1), 77-88.

Tang, J., Critchley, H. D., Glaser, D. E., Dolan, R. J., & Butterworth, B. (2006). Imaging informational conflict: A functional magnetic resonance imaging study of numerical Stroop. *Journal of Cognitive Neuroscience*, *18*(12), 2049-2062.

Thioux, M., Pesenti, M., De Volder, A., & Seron, X. (2001). Category-specific representation and processing of numbers and animal names across semantic tasks: A PET study. *Neuroimage*, *13*(6), 617.

Thompson, J. C., Abbott, D. F., Wheaton, K. J., Syngeniotis, A., & Puce, A. (2004). Digit representation is more than just hand waving. *Cognitive Brain Research*, *21*(3), 412-417.

Troiani, V., Peelle, J. E., Clark, R., & Grossman, M. (2009). Is it logical to count on quantifiers? Dissociable neural networks underlying numerical and logical quantifiers. *Neuropsychologia*, *47*(1), 104-111.

Tschentscher, N., Hauk, O., Fischer, M. H., & Pulvermüller, F. (2012). You can count on the motor cortex: finger counting habits modulate motor cortex activation evoked by numbers. *Neuroimage*, *59*(4), 3139-3148.

Venkatraman, V., Ansari, D., & Chee, M. W. (2005). Neural correlates of symbolic and non-symbolic arithmetic. *Neuropsychologia*, *43*(5), 744-753.

Vogel, S. E., Grabner, R. H., Schneider, M., Siegler, R. S., & Ansari, D. (2013). Overlapping and distinct brain regions involved in estimating the spatial position of numerical and non-numerical magnitudes: an fMRI study. *Neuropsychologia*, *51*(5), 979-989.

Wei, W., Chen, C., Yang, T., Zhang, H., & Zhou, X. (2014). Dissociated neural correlates of quantity processing of quantifiers, numbers, and numerosities. *Human brain mapping*, *35*(2), 444-454.

Wood, G., Nuerk, H. C., Moeller, K., Geppert, B., Schnitker, R., Weber, J., & Willmes, K. (2008). All for one but not one for all: How multiple number representations are recruited in one numerical task. *Brain research*, *1187*, 154-166.

Zago, L., Petit, L., Turbelin, M. R., Andersson, F., Vigneau, M., & Tzourio-Mazoyer, N. (2008). How verbal and spatial manipulation networks contribute to calculation: an fMRI study. *Neuropsychologia*, *46*(9), 2403-2414.

Zhang, H., Chen, C., & Zhou, X. (2012). Neural correlates of numbers and mathematical terms. *NeuroImage*, *60*(1), 230-240.

Zhou, X., Chen, C., Zhang, H., Xue, G., Dong, Q., Jin, Z., ... & Chen, C. (2006). Neural substrates for forward and backward recitation of numbers and the alphabet: A close examination of the role of intraparietal sulcus and perisylvian areas. *Brain research*, *1099*(1), 109-120.
